# Epidemiology of Musculoskeletal Injury during Racing on New Zealand Racetracks 2005–2011

**DOI:** 10.3390/ani7080062

**Published:** 2017-08-11

**Authors:** Charlotte Bolwell, Chris Rogers, Erica Gee, Wayne McIlwraith

**Affiliations:** 1 Equine Research Centre, Institute of Veterinary, Animal and Biomedical Sciences, Massey University, Private Bag 11-222, Palmerston North 4442, New Zealand; c.w.rogers@massey.ac.nz (C.R.); e.k.gee@massey.ac.nz (E.G.); 2 Equine Orthopaedic Research Center, Colorado State University, Fort Collins, CO 80523, USA; wayne.mcilwraith@colostate.edu

**Keywords:** horse, racehorse, incidence rates, cardiac, respiratory, fatalities

## Abstract

**Simple Summary:**

There is currently limited information on the types, or risk, of injuries occurring for horses racing in flat races in New Zealand. Race reports and records from six racing seasons were used to determine the reasons why horses failed to finish a race. In total, 544 horses failed to complete a race, of which 177 were due to veterinary events. Most of the veterinary events that occurred during a race were classed as musculoskeletal injuries (136/177; 77%). The rate of musculoskeletal injuries during a race, 0.72 per 1000 starts, was lower than the rates reported for other racing jurisdictions. The condition of the track and the distance of the race were associated with the rate of musculoskeletal injury during a race. There may be differences in the training programmes and racing schedules for horses in the southern hemisphere, which may have contributed to the low rates reported in this study.

**Abstract:**

The objective of the study was to determine the incidence of veterinary events that resulted in a horse failing to finish a race and identify risk factors for musculoskeletal injury (MSI) during a race. Data were obtained on Thoroughbred flat race starts in New Zealand between 1 August 2005 and 31 July 2011 (six racing seasons). Stipendiary Steward’s reports were key-word searched to identify veterinary events that prevented a horse from finishing a race. Race data were used calculate the incidence of veterinary events per 1000 horse starts and Poisson regression was used to investigate risk factors for MSI. There were 188,616 race starts and 177 reported veterinary events. The incidence of MSI on race day was 0.72 per 1000 starts, whilst the incidence of respiratory events was 0.21 per 1000 starts. The rate of MSI was significantly lower on ‘dead’ and ‘slow’ tracks compared with ‘good’ tracks and significantly greater in longer races (≥1671 m) compared with races of ≤1200 m. The rate of MSI during flat races in New Zealand appears lower than that reported worldwide, which may be due to the management and training of horses in New Zealand or differences in case definitions used in comparable studies.

## 1. Introduction

Over the last few decades, numerous epidemiological studies have focused on quantifying the incidence rates and risk factors for non-fatal and fatal race-day injuries in Thoroughbred racehorses worldwide. Internationally, the incidence of fatalities ranges from 0.4 per 1000 starts [1] to 1.9 per 1000 starts [2] and the incidence of musculoskeletal injury (MSI) reported on race day ranges from 2.1 per 1000 starts [3] to 4.4 per 1000 starts [4]. Previous studies have reported the incidence rates and risk factors for MSI occurring during training in New Zealand [5,6]. However, no studies have investigated the incidence of musculoskeletal events occurring on race day during flat races in New Zealand. 

A previous study reported a failure to finish rate of 2.8 per 1000 starts within flat racing in New Zealand [7]. Failure to finish data represented a broad category of events (pulled-up/fall/lost rider), that included MSI, which prevented a horse from completing the race. Risk factors for the broad categories of failure to finish included racing and environmental factors such as season, race distance, and ratings bands [7]. Tanner et al. 2016 [7] reported that approximately half of the failure to finish events were due to MSI occurring during a race. To date, the specific veterinary events resulting in a horse failing to finish a race have not been reported. 

As part of the rules of racing in New Zealand a Stipendiary Steward’s report is produced for each race detailing any events that occurred during the race. Historically, Thoroughbred racing administered their own Stipendiary Steward’s reports and race-day regulatory control, until 2011 when the Racing Integrity Unit (RIU) was established [8]. Whilst Stipendiary Steward’s reports may lack detailed veterinary diagnoses found in international databases of racing injuries [2], they can be used to determine the incidence of race events that prevent the horse from finishing a race [7] and provide descriptions of broad categories of veterinary events and injuries sustained to horse and rider during the race [8]. Such data can be used to describe the incidence of horses failing to finish a race due to injury and the risk factors for race-day MSI.

The objectives of the study were to: (1) describe the distribution and determine the incidence of veterinary events that resulted in a horse failing to finish a race; and (2) identify risk factors for MSI that resulted in a horse failing to finish a race.

## 2. Materials and Methods

A retrospective cohort study was conducted, using data from all Thoroughbred flat race starts in New Zealand between 1 August 2005 and 31 July 2011 (six racing seasons). The study utilised data from a previous study describing the broad failure to finish events (pulled-up/fall/lost rider) [7]. Briefly, Stipendiary Steward’s reports and race data were provided by New Zealand Thoroughbred Racing (NZTR). Race data were provided in a Microsoft Excel file and included race date, race track, race number, race class, race distance, track condition (or ‘going’), penetrometer reading, horse name, horse age, horse gender, finishing position in race (used to identify horses that failed to finish), barrier draw (position in the starting gates), carded weight (weight allocated by race handicapper), carried weight (carded weight less any apprentice weight allowance), and domestic rating (analogous to the British horse racing “official rating” system). Stipendiary Steward’s reports were provided as Microsoft Word documents.

Horses that were previously coded as a failure to finish by Tanner et al. [7] were identified from the dataset and the Stipendiary Steward’s report for the corresponding race was then keyword searched using horse and race information in order to identify the horse recorded as a failure to finish. The failure to finish event as reported on the Stipendiary Steward’s report was then categorised based on the terms listed in Table 1. In many cases the Stipendiary Steward’s report descriptions of the event were broadly described, preventing a detailed analysis of the type and location of the injuries sustained (Table 1). The descriptions of the horse related race events are provided by the on-course veterinarian, who is in attendance at any race to provide first aid when needed. 

The types of veterinary events reported in each category and the number of fatal events were summarised as counts and percentages. The incidence of veterinary events listed in Table 1 and the incidence of MSI fatalities were calculated and reported as events per 1000 horse starts. A horse could contribute several starts and more than one veterinary event over the study period. Horses that were withdrawn before starting a race (entering the starting gates and gates released) were excluded.

Race data were screened for errors and structured for analysis as described previously. Briefly, the continuous variables that were categorised into groups included: weight carried (quartiles), race distance (quartiles), and field size (number of starters in the race) (quartiles). New variables were created for race year, season (spring, summer, autumn, winter), field size, and whether or not the jockey had an apprentice allowance. Ratings were categorised based on the ratings bands recognised by the New Zealand handicapping system which is analogous to the rating system used by the British Horse Racing Board [7]. 

Poisson regression was used to estimate incidence rate ratios (IRR) with 95% CI for race exposure variables and the outcome MSI. A null model was fitted with horse as a random effect to check for clustering of veterinary events at the horse level. Results showed no significant clustering of the outcomes among horses, leading to subsequent models including only fixed effects. Variables showing association with the outcome (*p* < 0.2) in univariable analysis were analysed in multivariable regression models fitted in a backwards step-wise fashion. To determine the best fit for the continuous variables in the multivariable models, the IRR for the categorical and continuous variables were compared and a likelihood ratio test was used to compare a model with the continuous form and a model with the corresponding categorical variable. During the multivariable modelling variables were retained in model using a likelihood ratio *p*-value *p* ≤ 0.05 or if there was evidence of confounding. A postestimation test was performed to assess the final model fit using the Pearson chi-squared goodness-of-fit test. All analyses were conducted in Stata version 12 (College Station, StataCorp LP, TX, USA).

## 3. Results

During the six racing seasons, there were 188,616 race starts for 16,646 individual horses. There were 544 failed to finish events, of which 177 (33%) were classed as veterinary events and 48% (85/177) of these were fatalities. Of the veterinary events reported, 136 (77%) were due to MSI and 41 (23%) were cardiac and respiratory events. The incidence of MSI on race day was 0.72 per 1000 starts, whilst the incidence of cardiac and respiratory events was 0.21 per 1000 starts. 

Of the MSI events, 91 (67%) were reported as fractures, 10 (7%) were reported tendon or ligament injuries, 10 (7%) were reported as lameness, and 7 (5%) were reported as soft tissue injuries; 18 (13%) MSI events were undefined. Only one horse had multiple veterinary events (*n* = 2), of which one was lameness and one was tendon and ligament. The incidence of fractures, tendon or ligament injuries, and lameness, and soft tissue injuries was 0.48, 0.05, and 0.04 per 1000 starts, respectively. Of the MSI events 75 (55%) were forelimb, 33 (22%) were hindlimb, 1 (1%) was both fore and hindlimb, 9 (7%) were pelvic and 19 (14%) were non-defined. A total of 57% (78/136) of MSI were fatalities, whilst 81% (72/89) of fractures were fatalities. The incidence rate for race-day MSI fatalities was 0.41 per 1000 starts. 

Over half of the cardiac and respiratory events were recorded as epistaxis (23/41; 56%), five were recorded as atrial fibrillation, five were recorded as fatal haemorrhage, four were recorded as elevated heart rate, three events were recorded as ruptured aorta, and one event was recorded as respiratory distress. A total of seven (17%) cardiac and respiratory events were recorded as fatal.

Results of the univariable Poisson regression analysis for MSI are presented in Table 2. Race year, sex of horse, age of horse, apprentice allowance, barrier draw, and race number (order of race at the race meeting) were not significantly associated with MSI at the univariable level. Season, track condition, race distance, and weight carried were associated with MSI at *p* < 0.20 and were included in the multivariable model. 

The results of the multivariable Poisson regression model of variables significantly associated with MSI are presented in Table 3. Race distance and track condition were significantly associated with MSI. After adjusting for race distance, the rate of MSI was significantly lower on ‘dead’ and ‘slow’ tracks compared with ‘good’ tracks. After adjusting for track condition, the rate of MSI was significantly greater for horses in longer races (≥1671 m) compared with horses in races of ≤1200 m (Table 2). There were no significant interactions in the final model. The Pearson goodness of fit statistic for the final model was *p* = 0.56, indicating no evidence of poor model fit.

## 4. Discussion

This is the first study to report on the incidence of failure to finish a race due to MSI in flat races in New Zealand and provide an assessment of the risk factors for MSI during a race. The incidence of MSI during flat races in New Zealand appears to be low when compared with international data. Recent work from the UK reported a race-day MSI incidence of 2.1 per 1000 starts [3], whilst Cohen and others [4] reported a MSI rate of 4.1 per 1000 starts in Kentucky. Similarly, the rate of MSI fatalities during a race was lower than that previously reported in the USA (1.9 per 1000 starts) [2] and the UK (0.7 per 1000 starts) [3]. It is unlikely that the incidence of MSI during a race was underestimated in this study, as failure to finish a race represents a key event that prevented a horse from completing the race [7], which was subsequently recorded on official race-day records. Due to the nature of the recording pre-2011 (when the RIU was established) it is possible that the rate of fatalities may be underestimated in the current study. However, the rate of fatalities reported in this study was the same as that previously reported in a study of race-day fatalities in Victoria, Australia [1], a racing jurisdiction with similar structures and levels of reporting as found in New Zealand. 

The lower incidence rates reported here may relate to the training and management of racehorses in New Zealand. Previous studies of racehorses in training identified associations between exercise distances accumulated in training and breaks from training and various measures of training and racing performance [9,10,11]. Specifically, horses with a voluntary or involuntary interruption to training before their first trial were less likely to trial, resulting in fewer overall trial starts, which was associated with a reduced chance of a race start [9]. It may that the trainers’ perception of the horse’s ability and soundness to trial and race, with trainer deciding to retire a horse rather than continue to train and race it. Therefore, it is possible that the flat racing population of horses in New Zealand may be likened to the previously described “healthy horse effect” or survival bias [12] and the lower rates of MSI observed during races. Similarly, many studies have reported associations between exercise distances accumulated in training, prior racing history and time between races, and the risk of MSI and fatalities in training and racing [5,12,13]. However, these relationships are known to be complex and are likely to vary with different case definitions investigated. Training data were not available for the cohort of horses used in the current study but further work to address the relationship between exercise history and the rates of MSI during racing is required. 

In addition to potential differences in training and racing schedule, the track surface used for races in New Zealand compared to other racing jurisdictions may explain the low rate of MSI observed in this study. Racing in New Zealand is conducted on grass tracks [14], whereas races in the USA are most commonly run on dirt tracks [15], which have higher rates of injuries compared to synthetic tracks. However, given that the rates of injuries on turf tracks in the USA are higher than those reported for synthetic tracks [15], it is likely that other factors in addition to track surface contribute to the higher rates observed internationally. Differences in the rules of racing, for example, permit the use of therapeutic medications in the USA [16], and the structure and type of racing across racing jurisdictions [7] may also contribute to the variation in rates reported worldwide. Given this, there is a need for a collaborative approach to determine regional similarities or differences that contribute to the rate of racing MSI. 

Only two exposure variables were found to be associated with the incidence of MSI during a race in the multivariable modelling. Longer race distances were found to be a risk factor for MSI, in agreement with results of studies investigating racing fractures and fatalities [17,18]. An Australian study reported increased odds of racing fatalities, of which most were due to MSI, for every additional furlong raced [17]. The authors of that study suggested the increased risk was likely due to increased exposure time for an injury to occur, and the possibility of more fatigued horses in longer races [17]. The race distances in Australia are similar to those of New Zealand, with a maximum distance of between 3200–3600 m. 

The results showed a lower risk of MSI on “dead” and “slow” tracks when compared with races run on “good” tracks. An increased incidence of MSI or risk of fatalities or fractures on “fast” or “good/firm” tracks compared to “heavy/soft” tracks has been reported in Australia [17] and the UK [3,18]. Within New Zealand, there appears to be an active programme by track managers to avoid fast tracks with watering of tracks in summer and extensive drainage in winter to improve the consistency of the going [19]. This may explain the low number of fast tracks observed in this dataset, which may have been a contributor to the apparent inability of the present study to identify fast tracks as a risk factor for MSI despite most of the MSI being due to fracture. The avoidance of the harder, less compliant track conditions rated as “fast” by New Zealand track managers is of interest and the drivers behind this decision require further examination.

Most of the MSI veterinary events in this study were reported as fractures, with smaller numbers of tendon and ligament and lameness and soft tissue injuries reported. A previous study reported 42% and 41% of retirements from racing in New Zealand were due to fracture or tendon injuries, respectively [11]. However, Perkins et al. [11] included injuries occurring in training making direct comparisons with the results of this study difficult. The incidence rates of tendon and ligament injuries and soft tissue injuries were lower than recently reported in a UK study of veterinary events in flat racing [3]. It is possible that the lower incidence of MSI reported in our study was due to differences in the inclusion criteria between the two studies. Our study focused on the events occurring during a race, rather than those that could be reported on race-day, as with the UK study [3], which may occur after a race has ended and the horse has crossed the finish line. Furthermore, the veterinary events occurring during a race are reported to the Stipendiary Stewards by the on-course veterinarian who may vary for each race meeting, which may have an impact on the diagnoses made and reported in the Stipendiary Steward’s report. It should also be noted that these diagnoses would be made with the diagnostics tools available on the racecourse, which may partly explain the broad categories reported to describe the veterinary events.

It is possible that the lower incidence rates reported here were due to missing or misclassification of data [11]. The UK study utilised detailed data where, although some misclassification may have occurred, rigorous electronic recording of veterinary information was enforced; often allowing the type of condition/injury and anatomical location to be described [3]. The low number of detailed veterinary events reported resulted in a lack of power to investigate specific case definitions such as fractures and tendon and ligament injuries in the current study. Whilst the use of broad definition of MSI in this study is likely to have minimised the potential for misclassification of the outcome, it is important to highlight this common limitation of retrospective studies. Therefore, more robust data are required to allow a detailed analysis of the incidence of different types of veterinary events occurring during flat races, in New Zealand, and the associated risk factors. 

In 2011, the RIU took over the monitoring and management of the Stipendiary Steward’s reports in New Zealand [8]. A previous study indicated that there were some improvements in the quality of the reporting of race events, associated with jockey falls, on Stipendiary Steward’s reports after the RIU was established [8]. Such information included more consistent recording of whether a jockey received medical treatment [8]. Whether similar improvements would be noted with regards to the level of detail recorded for horse events is yet to be determined. In 2014, an incident report form was implemented by NZTR based on that used in Australia, which will provide more standardised data on the veterinary events occurring on race day. Due to the low incidence rates reported in this study, data will be required from a number of racing seasons post-2014 in order to provide robust data for further analysis.

## 5. Conclusions

Overall, the rate of MSI reported during a race was lower than international figures for race-day MSI. A detailed analysis of a range of musculoskeletal case definitions was prevented in this study due to a lack of consistent reporting of veterinary diagnosis on the Stipendiary Steward’s reports. To obtain a better understanding of the injuries associated with racing in New Zealand, there is a need for greater robustness and standardisation of descriptors used to record veterinary events occurring during a race; including the site on the horse, type of injury, and location on track where the event occurred. This is urgently required in order to facilitate future studies to understand the characteristics of the low rate of racing MSI in New Zealand.

## Figures and Tables

**Table 1:** Categories and definitions used to describe failure to finish veterinary events reported by Stipendiary Stewards occurring during Thoroughbred flat races in New Zealand (2005–2011).

Category	Definition
Musculoskeletal injury	A failure to finish event due to an injury to the muscular or skeletal system. Included terms such as: fracture, injury, broke down, sore, lameness, ruptured, torn, lacerations, damaged, broke
Fracture	A failure to finish event due to a fracture. Included any event where fracture was specifically stated.
Tendon and ligament	A failure to finish event due to a tendon or ligament injury. Included terms such as tendon severed, ruptured suspensory, injury tendon, broke down right tendon
Lameness	A failure to finish event due to lameness or soreness. Included terms such as sore behind, unsound, sore, lame
Soft Tissue	A failure to finish event due to a muscle or skin injury. Included terms such as pulled muscle, muscle soreness, torn muscle, cut leg
Undefined	A failure to finish event due to an undefined MSI. Included terms such as injury hind quarters, injury left leg, injuries lower limb, broke down, fell and euthanased
Cardiac and respiratory	A failure to finish event that was a result of a respiratory or cardiovascular event that was not influenced by injury. Such as epistaxis, atrial fibrillation, elevated heart rate, heart fibrillation, ruptured aorta, heart fibrillation, bled, ruptured, haemorrhage, respiratory distress

**Table 2:** Univariable Poisson regression for musculoskeletal injuries occurring during Thoroughbred flat races in New Zealand (2005–2011) (*n* = 136) ^a^.

Variable	Level	No. of Starts	No. of Musculoskeletal Injuries	IRR	95% Confidence Interval	*p* Value	Wald *p* Value
Race year	2005/06	29,751	18	-	-	-	0.73
	2006/07	30,574	27	1.46	0.80–2.65	0.21	
	2007/08	31,276	23	1.21	0.65–2.25	0.53	
	2008/09	33,061	20	0.99	0.52–1.89	1.00	
	2009/10	32,349	22	1.12	0.60–2.09	0.71	
	2010/11	31,605	26	1.36	0.74–2.48	0.32	
Season	Spring	49,620	40	-	-	-	0.13
	Summer	52,647	45	1.06	0.69–1.62	0.79	
	Autumn	48,484	24	0.61	0.37–1.02	0.06	
	Winter	37,865	27	0.88	0.54–1.44	0.62	
Sex	Male	104,605	73	-	-	-	
	Female	84,011	63	1.07	0.77–1.50	0.68	0.68
Track condition	Fast	5478	6	-	-	-	0.04
	Good	73,231	67	0.83	0.36–1.92	0.67	
	Dead	44,481	24	0.49	0.20–1.20	0.12	
	Slow	32,310	15	0.42	0.16–1.09	0.08	
	Heavy	33,116	24	0.66	0.27–1.62	0.37	
Age (year)	2	6072	3	-	-	-	0.52
	3	43,228	26	1.22	0.36–4.02	0.328	
	4	56,374	38	1.36	0.42–4.42	0.129	
	5	42,439	33	1.57	0.48–5.13	0.234	
	6+	40,503	36	1.80	0.55–5.84	0.58	
Apprentice allowance	No	144,005	102	0.93	0.63–1.37	0.71	0.71
	Yes	44,611	34	-	-	-	
Race distance	≤1200 m	49,554	25	-	-	-	0.002
	1201–1400 m	47,914	27	1.12	0.65–1.92	0.69	
	1401–1670 m	44,587	31	1.38	0.81–2.33	0.23	
	≥1671 m	46,561	53	2.26	1.40–3.63	0.001	
Weight carried	46–54.5 kg	55,382	52	-	-	-	0.15
	54.6–55.5 kg	40,677	25	0.65	0.41–1.05	0.08	
	55.6–56.9 kg	38,547	27	0.75	0.21–1.19	0.22	
	57–76 kg	54,010	32	0.63	0.41–0.98	0.04	
Rating bands	50–54	46,817	29	-	-	-	0.71
	55–65	45,695	38	1.34	0.82–2.18	0.23	
	66–75	57,524	38	1.07	0.66–1.73	0.79	
	76–85	21,639	18	1.34	0.75–2.42	0.33	
	86–115	16,941	13	1.24	0.64–2.38	0.52	
Field size	3–9	43,560	26	-	-	-	0.71
	10–11	42,003	31	1.24	0.73–2.08	0.42	
	12–13	45,245	36	1.33	0.80–2.21	0.26	
	14–18	57,808	43	1.25	0.76–2.03	0.38	
Barrier	1–3	51,029	31	-	-	-	0.67
	4–6	50,625	41	1.33	0.83–2.12	0.23	
	7–9	44,443	33	1.22	0.75–1.99	0.42	
	10–21	42,519	31	1.20	0.73–1.97	0.47	
Race number	1	16,942	10	-	-	-	0.27
	2	18,389	11	1.01	0.43–2.37	0.98	
	3	18,596	10	0.91	0.38–2.19	0.83	
	4	18,840	10	0.90	0.37–2.16	0.81	
	5	19,364	23	2.01	0.96–4.23	0.06	
	6	19,732	15	1.29	0.58–2.87	0.53	
	7	19,925	13	1.10	0.48–2.52	0.81	
	8	20,507	16	1.32	0.60–2.91	0.49	
	9	17,487	18	1.74	0.80–3.78	0.16	
	10+	18,834	10	0.90	0.37–2.16	0.81	

^a^ Showing incidence rate ratios (IRR) for all Thoroughbred flat race starts (*n* = 188,616) in the 2005/06–2010/11 racing years.

**Table 3:** Results of multivariable Poisson regression model of the variables significantly associated with musculoskeletal injury occurring during Thoroughbred flat races in New Zealand (2005–2011) (*n* = 136) ^a^.

Variable	Level	No. of Starts	No. of Musculoskeletal Injuries	IRR	95% Confidence Interval	*p* Value	LRT *p* Value
Total		188,615	136				
Track condition	Fast	5478	6	1.17	0.51–2.69	0.71	0.002
	Good	73,231	67	-	-	-	
	Dead	44,481	24	0.59	0.34–0.95	0.03	
	Slow	32,310	15	0.51	0.29–0.90	0.02	
	Heavy	33,116	24	0.80	0.50–1.3	0.34	
Race distance	≤1200 m	49,554	25	-	-	-	0.04
	1201–1400 m	47,914	27	1.11	0.64–1.91	0.71	
	1401–1670 m	44,587	31	1.35	0.80–2.29	0.26	
	≥1671 m	46,561	53	2.21	1.38–3.57	0.001	

^a^ For all Thoroughbred flat race starts (*n* = 188,616) in the 2005/06–2010/11 racing years.
